# First Report on Microbial-Derived Polydeoxyribonucleotide: A Sustainable and Enhanced Alternative to Salmon-Based Polydeoxyribonucleotide

**DOI:** 10.3390/cimb47010041

**Published:** 2025-01-09

**Authors:** Doobyeong Chae, Sae-Woong Oh, Yoon-Seo Choi, Dae-Jung Kang, Chun-Woong Park, Jongsung Lee, Won-Sang Seo

**Affiliations:** 1 Department of Cosmetics Industry, Graduate School, Chungbuk National University, Cheongju 28160, Republic of Korea; enqud2@gmail.com; 2 Molecular Dermatology Laboratory, Department of Integrative Biotechnology, College of Biotechnology and Bioengineering, Sungkyunkwan University, Suwon 16419, Republic of Korea; hanzeeoo@skku.edu; 3 Graduate School-Interdisciplinary Program in Biocosmetics, Sungkyunkwan University, Suwon 16419, Republic of Korea; eveelf@g.skku.edu; 4 MNH Bio Co., Ltd., Hwaseong 18469, Republic of Korea; djkang@mnhbio.com; 5 Department of Manufacturing Pharmacy, College of Pharmacy, Chungbuk National University, Cheongju 28160, Republic of Korea; cwpark@chungbuk.ac.kr; 6 COSLINK, Songpa-gu, Seoul 05819, Republic of Korea

**Keywords:** PDRN (polydeoxyribonucleotide), microbial-derived PDRN, probiotics, wound healing, anti-inflammatory effects

## Abstract

Polydeoxyribonucleotide (PDRN) has emerged as a potent bioactive compound with proven efficacy in wound healing, tissue regeneration, and anti-inflammatory applications and is predominantly derived from salmonid gonads. However, this study presents a groundbreaking advancement by successfully extracting and characterizing PDRN from microbial sources, specifically *Lactobacillus rhamnosus*, marking the first report to utilize microbial-, biome-, or *Lactobacillus*-derived PDRN (L-PDRN). The findings demonstrate the enhanced biological properties of L-PDRN over traditional salmon-derived PDRN across several assays. L-PDRN exhibited superior antioxidant activity, with significantly higher SOD-like and DPPH radical scavenging activities compared to PDRN, particularly at higher concentrations. In wound-healing assays, L-PDRN demonstrated superior efficacy in promoting cell migration and wound closure, even under inflammatory conditions induced by tumor necrosis factor (TNF-α). Additionally, L-PDRN demonstrated the potential for enhanced immunostimulatory effects under non-inflammatory conditions while maintaining anti-inflammatory properties under lipopolysaccharide (LPS) stimulation. Electrophoretic analysis revealed that L-PDRN consists of smaller DNA fragments (under 100 bp) compared to salmon-derived PDRN (200–800 bp), suggesting greater bioavailability and skin absorption. Mechanistic studies confirmed that L-PDRN activates the focal adhesion kinase (FAK) and protein kinase B (AKT) signaling pathway through the A2A receptor, similar to PDRN, while also engaging alternative pathways for p38 and ERK phosphorylation, highlighting its signaling versatility. This study underscores the potential of L-PDRN as a multifunctional and sustainable alternative to salmon-derived PDRN, offering enhanced bioactivity, scalability, and environmental benefits. The novel approach of utilizing microbial-derived PDRN opens new avenues for therapeutic applications in oxidative stress management, tissue regeneration, and immune modulation, paving the way for a paradigm shift in PDRN sourcing and functionality.

## 1. Introduction

Polydeoxyribonucleotide (PDRN) is a linear polymer of DNA, typically derived from the gonads of salmon trout. It consists of a mixture of deoxyribonucleotides with chain lengths ranging from 80 to 2200 base pairs and a molecular weight between 50 and 1500 kDa. These nucleotides include adenosine, which has been shown to exhibit various pharmacological effects by binding to adenosine receptors [1,2,3]. Adenosine plays a key role in numerous physiological and pathological processes, mediated by four distinct receptors: A1, A2A, A2B, and A3. Among these, the A2A receptor is primarily involved in regulating inflammation, ischemia, cell proliferation, and angiogenesis [4]. PDRN typically acts as an agonist for the A2A receptor, modulating several intracellular activities [4,5].

PDRN has been shown to be effective in promoting wound healing [4], tissue regeneration, and reducing inflammation, as well as regulating cytokine signaling pathways involved in collagen production [6,7]. Additionally, PDRN is known to enhance corneal epithelial regeneration and alleviate pain and disability in patients with rotator cuff disease [8,9,10]. It also improves wound closure and promotes re-epithelialization in patients with refractory diabetic foot ulcers, being particularly effective in skin wound regeneration [11]. PDRN stimulates wound healing by acting as a growth promoter and chemokine, promoting the proliferation of epithelial cells at the wound site [12,13].

Currently, PDRN is predominantly extracted from salmonid gonads (*Oncorhynchus mykiss* and *O. keta*). However, the limitations of seasonal availability, high production costs, and environmental sustainability have prompted investigations into alternative sources. Above all, salmon is a limited resource. If the demand for PDRN surges, alternatives will become essential.

Recent studies have highlighted plant-derived PDRN as a viable alternative to traditional sources like salmon. PDRN from Korean ginseng roots promotes skin cell regeneration and wound healing [14]. Other plant-derived PDRNs, such as those from mugwort and broccoli, have shown significant wound-healing effects in vitro [15]. Marine-derived PDRN sources beyond salmon, like the sea cucumber (*Apostichopus japonicus*), have demonstrated antioxidant properties and therapeutic potential [16]. PDRN has been successfully extracted from the seaweed *Porphyra* sp. (nori). A study on Nile tilapia (*Oreochromis niloticus*) showed that PDRN from *Porphyra* enhanced the healing of artificially induced skin wounds, highlighting its potential therapeutic applications in aquaculture [17].

Microbial sources offer significant advantages, such as rapid growth, year-round availability, and a lower environmental impact compared to plant or marine sources. Additionally, they can be cultivated artificially, reducing concerns about endangered species. However, research on microbial-derived PDRN is currently lacking, with most studies focusing on plant- and marine-derived alternatives. A patent application was filed claiming that DNA derived from *Leuconostoc*, rather than *Lactobacillus*, promotes wound healing. However, the application did not specifically mention PDRN, lacked experimental results and explanations regarding the A2A receptor-related mechanism, and provided insufficient details to support the claims. Consequently, the application was rejected both in South Korea and internationally [18]. Probiotics are GRAS (generally recognized as safe) microorganisms that are safe and free from issues for use in the human body. For this reason, probiotics are considered a good source of PDRN.

Probiotics are active microorganisms that provide beneficial effects to the host by modulating the composition of the microbiota in specific areas of the host’s flora [19]. Strategies leveraging the skin microbiome to improve skin health include the gut–skin axis theory, which highlights the impact of regulating the gut microbiome on the skin’s microbial balance [20]. Emerging evidence indicates that oral probiotics can play a role in managing skin photoaging by influencing gut–skin microbiota interactions. These probiotics help reduce oxidative stress, suppress inflammation, maintain immune balance, and protect the skin’s extracellular matrix [21,22,23].

The topical approach involves applying specific probiotic strains or elements directly to the skin to influence its microbiota. This method, gaining recognition for its intuitive appeal, aims to prevent and address skin photoaging by introducing cultured live bacteria in precise amounts. Originally proposed as a treatment for conditions like acne and seborrhea, this strategy seeks to restore a balanced skin microbiota and immune equilibrium [24,25].

However, investigations into the influence of probiotics on skin microflora and the subsequent effects on the skin are notably scarce. Probiotics can modulate the microbiome by inhibiting harmful microbes while promoting beneficial ones [26]. Probiotics have several beneficial effects on the skin and can help reduce the signs of skin aging caused by exposure to sunlight. Firstly, probiotics reduce oxidative stress levels, which are closely associated with the development of skin aging [21,27]. They enhance the activity of antioxidant enzymes, decrease the production of reactive oxygen species (ROS), and inhibit signaling pathways involved in the breakdown of collagen and the synthesis of matrix metalloproteinases (MMPs) [27,28]. This ultimately leads to a decrease in the damage to the skin caused by ROS and the aging process. Probiotics have an inhibitory effect on the inflammatory cascade, which is responsible for disrupting the skin barrier, increasing water loss through the skin, and accelerating the aging process. Probiotics can suppress the production of pro-inflammatory cytokines, regulate immune responses, and maintain immune balance [27,29]. They also help alleviate skin inflammation caused by exposure to ultraviolet [30] radiation, thereby preventing skin aging [31]. Probiotics inhibit the remodeling of the extracellular matrix (ECM), which is a critical process affected by UV exposure [21,22]. They regulate the expressions of MMPs and TIMPs (tissue inhibitors of metalloproteinases), thereby reducing the degradation of collagen and elastin. This results in improved skin structure and elasticity, preventing issues such as roughness, sagging, and wrinkling [22].

Topical probiotics are being studied for their potential benefits in skincare, specifically in addressing photoaging and skin aging [32]. Research suggests that they can help slow down the aging process, reduce oxidative stress, and improve the skin’s barrier function [33]. Studies have explored the use of probiotics fermented with plant extracts and *Nitrosomonas eutropha* to treat wrinkles and improve hyperpigmentation, showing promising results [34,35]. However, there are not many cases where the effectiveness of probiotics alone has been confirmed.

Interestingly, most probiotics currently under investigation are not directly associated with human skin. As the skin ages, it not only experiences a reduction in collagen and elastin but also, according to recent studies, a decline in *Lactobacillus* on its surface [36]. Although some research has been conducted on common *Lactobacillus* strains, studies specifically focused on *Lactobacillus* isolated from human skin remain quite limited, including those involving the authors of this study [37,38]. While research on skin-derived *Lactobacillus* has recently started, investigations into *Lactobacillus*-derived DNA or PDRN have yet to begin.

This study focuses on microbial-derived PDRN extracted from *Lactobacillus rhamnosus*, evaluating its efficacy in anti-inflammatory and wound-healing applications compared to traditional salmon-derived PDRN. The purpose of this study is to explore the advantages of novel PDRN beyond the homology of salmon-derived PDRN. This study is particularly significant as it is the very first to investigate the properties of PDRN derived from microbial, biome, and *Lactobacillus* sources.

## 2. Materials and Methods

### 2.1. Bacterial Strain and Culture

The culturing process for *Lactobacillus rhamnosus* (COSLINK Management Number CL-315 or MNH Bio Management Number UBC-U937) was initiated with a primary seed culture in De Man–Rogosa–Sharpe agar (MRS) media. This strain, *Lactobacillus rhamnosus*, was isolated from *Glycine soja*, the wild ancestor of soybean (*Glycine max*), which is known to originate from Korea [39]. The culture was incubated at optimal conditions for bacterial growth for 18 h. Upon completion, 5% of the culture volume was transferred into fresh MRS media to conduct a secondary seed culture, which was incubated for an additional 8 h. Typically, MRS badges contain animal-derived ingredients. However, testing with a vegan-derived MRS, free of these ingredients, showed no significant differences in performance.

Following the secondary seed culture, 5% of the culture volume was again transferred into fresh MRS media for the main culture. This main culture was incubated for 18 h to achieve sufficient bacterial growth. The cultured bacterial cells were then harvested by centrifugation, separating the pellet from the supernatant. To ensure purity, the collected cell pellet was washed three times with saline solution to remove any extracellular components, resulting in purified bacterial cells for further processing.

### 2.2. PDRN Extraction Process

The purified bacterial cells were resuspended in triple-distilled water (DW) to prepare for PDRN extraction. The suspension was placed in a shaking incubator set at 45 °C and 100 rpm and allowed to react for 30 min. To extract PDRN, the bacterial cells underwent thermal pressure crushing under conditions of 121 °C and 0.2 MPa, ensuring effective cell lysis and PDRN release. DNA remains relatively stable at high temperatures under dry conditions, but it undergoes complete degradation at temperatures above 190 °C [40]. When exposed to high temperatures, DNA damage may prevent the appearance of specific bands during electrophoretic analysis. To ensure DNA integrity, this is meticulously monitored in each experiment.

The extract was immediately cooled to 4 °C, followed by centrifugation to separate the supernatant containing PDRN from the cellular debris. The supernatant was carefully collected for the subsequent purification steps.

### 2.3. PDRN Purification Process

The collected supernatant was filtered through a 0.45 μm PES filter to remove residual particles and debris. Sodium acetate was added to the filtered solution, adjusting its concentration to 0.3 M to facilitate ethanol precipitation. Ethanol was then introduced to the solution until the final concentration reached 70%, and the mixture was incubated at −20 °C overnight to allow PDRN to precipitate. The ethanol precipitation method at −20 °C is a commonly practiced approach for DNA precipitation [41]. After incubation, the precipitated mixture was centrifuged at 4 °C and 15,000 rpm for 20 min, and the supernatant was discarded. The pellet was washed with 70% ethanol, cooled to −20 °C, and centrifuged again under the same conditions. This washing and centrifugation step was repeated to ensure thorough purification of the pellet. The final purified pellet was collected for further processing.

### 2.4. Final Steps for PDRN Recovery

The purified pellet was dissolved in 1× TE buffer, ensuring complete dissolution of the PDRN. The dissolved PDRN solution was then filtered using a vacuum glass filtration system equipped with 0.45 μm and 0.20 μm PES membrane filters. The resulting PDRN solution, purified and filtered, was prepared for use in subsequent experiments. This method outlines a chemical-free, optimized approach to extract and purify PDRN from *Lactobacillus rhamnosus*, ensuring safety, efficacy, and structural integrity of the extracted DNA. Currently, the International Nomenclature of Cosmetic Ingredients (INCI) designates PDRN and L-PDRN as sodium DNA or hydrolyzed DNA. Additionally, the term salmon-derived PDRN refers to PDRN unless explicitly stated otherwise in the text.

### 2.5. Purity Assessment and Electrophoretic Analysis of L-PDRN

To confirm the successful isolation of L-PDRN and assess its DNA purity, we carried out the following experiment. A spectrophotometer was used to measure the optical density (OD) of L-PDRN at 260 nm wavelength and 280 nm wavelength, with distilled water serving as the blank. The DNA concentration was calculated by multiplying the OD at 260 nm wavelength by the dilution factor and 50 µg/mL. Purity was determined based on the ratio of the OD at 260 nm wavelength to that at 280 nm wavelength, where values between 1.8 and 2.0 were considered indicative of high-purity DNA.

To compare the DNA fragment sizes of L-PDRN and salmon-derived PDRN, an agarose gel electrophoresis was conducted. A 2% agarose gel was prepared and loaded with samples alongside a 100 bp DNA marker, and Dyne Loading STAR was used as the staining reagent. The electrophoresis was performed for 25 min.

### 2.6. SOD Assay

The activity of water-soluble antioxidants in suppressing the reactivity of the superoxide ion was measured using the SOD assay method by Marklund [42]. To do this, 0.1 mL of a pyrogallol solution (2 mM in 10 mM HCl) was combined with 0.9 mL of a 55.6 mM Tris-cacodylic acid buffer (TCB, pH 8.20) containing 1.1 mM DTPA, and the absorbance was monitored at 420 nm wavelength at 25 °C. The autoxidation rate of pyrogallol (control) was determined from the slope of the absorbance curve during the first minute of the reaction, with the control absorbance change measured at 0.02/min. To assess the SOD-like activity of the antioxidants, each compound (1 mM), which was dissolved in TCB, was added to the pyrogallol solution. The change in absorbance per minute in the presence of the antioxidant was compared to that of the control. The SOD-like activity (%) is calculated as follows:

SOD-like activity (%) = ((Absorbance of control − Absorbance of sample)/Absorbance of control) × 100.

### 2.7. DPPH Assay

The hydrogen atom or electron donation capabilities of certain pure compounds were assessed by measuring the decolorization of a purple methanol solution containing the stable DPPH radical. This spectrophotometric assay utilizes the stable radical 1,1-diphenyl-2-picrylhydrazyl (DPPH•) as the reagent [43]. This DPPH test was carried out following the modified method of Blois [44]. After preparing the samples and the positive control (ascorbic acid) by diluting them to different concentrations, 12.5 µL of the sample was added to 50 µL of ethanol. Then, 62.5 µL of 0.1 mM 2,2-Diphenyl-1-picrylhydrazyl (DPPH) was added, and the mixture was allowed to react for 30 min at 4 °C in the dark. The absorbance was measured at 520 nm wavelength to determine the concentration of DPPH radicals. The DPPH radical scavenging activity (%) is calculated as follows:

DPPH radical scavenging activity (%) = ((Absorbance of control − Absorbance of sample)/Absorbance of control) × 100.

### 2.8. Anti-Inflammatory Assay

The macrophage cell line used was RAW 264.7 cells (5 × 10^4^ cells/well), and the samples were treated at various concentrations. The NO measurement assay was conducted as follows. The NO (nitric oxide) measurement assay was performed using the Griess reagent method. After treating RAW 264.7 cells with the samples at various concentrations, the cells were incubated for 24 h at 37 °C in a humidified atmosphere containing 5% CO_2_. Following incubation, 100 µL of the culture supernatant was collected from each well and mixed with an equal volume of Griess reagent (1% sulfanilamide and 0.1% N-(1-naphthyl) ethylenediamine dihydrochloride in 2.5% phosphoric acid). The reaction mixture was incubated at room temperature for 10 min, allowing the formation of a pink azo dye. The concentration of lipopolysaccharide (LPS) used for induction is 1 µg/mL.

The absorbance of the mixture was then measured at 540 nm wavelength using a microplate reader. The concentration of NO was calculated based on a standard curve prepared using sodium nitrite solutions of known concentrations.

### 2.9. Wound-Healing Assay

The experiment aimed to investigate the regenerative effects of PDRN on scratched cell surfaces. To begin, HaCaT cells were seeded in a 24-well plate at a density of 1 × 10^5^ cells per well and cultured for 24 h in a humidified atmosphere containing 5% CO_2_ at 37 °C. This allowed the cells to grow and reach full confluency, forming a uniform monolayer in each well.

Once the monolayer was established, the cells were stimulated with tumor necrosis factor (TNF-α) to simulate an inflammatory environment. To induce a wound, a scratch was made across the cell monolayer using a sterile 1 mL yellow pipette tip. The scratch created a gap or “wound” area devoid of cells, mimicking an injury on the surface.

Following the scratch, the cells were treated with experimental PDRN samples at varying concentrations. The control group was treated with PBS (phosphate-buffered saline) as a negative control. After treatment, the plates were incubated, and the wound area was observed at two time points: 0 h (immediately after the scratch) and 18 h post-treatment.

To assess the regenerative effect, images of the scratched areas were captured using an inverted microscope at both time points. The images were then analyzed using the ImageJ software to measure the wound area in each well. The percentage of wound closure over 18 h was calculated as a measure of the cell migration and regenerative effect of the PDRN treatment compared to the PBS-treated control group.

### 2.10. Western Blotting

The immortalized keratinocyte HaCaT cells, maintained at Sungkyunkwan University, were used for the experiments. Cells (2 × 10^5^ per 60 mm culture dish) were treated with L-PDRN and 3,7-dimethyl-1-propargylxanthine (DMPX) at the indicated concentrations for 60 min. Subsequently, the cells were incubated for 24 h at 37 °C in a humidified atmosphere containing 5% CO_2_. After incubation, the cells were lysed in RIPA buffer (Cell Signaling Technology, Danvers, MA, USA), and protein concentrations were determined using a BCA assay (ThermoFisher Scientific, Waltham, MA, USA) according to the manufacturer’s instructions. A total of 20 µg of protein lysate was separated by SDS-PAGE and transferred onto a PVDF membrane (Bio-Rad, Hercules, CA, USA). Primary antibodies were diluted as follows and incubated overnight at 4 °C in a shaking incubator: phosphorylated focal adhesion kinase (FAK) and total FAK (1:1000; Cell Signaling Technology, Danvers, MA, USA), phosphorylated extracellular signal-regulated kinase (ERK)1/2 and total ERK1/2 (1:1000; Cell Signaling Technology, Danvers, MA, USA), phosphorylated protein kinase B (AKT) and total AKT (1:1000; Cell Signaling Technology), β-actin (1:10,000; Sigma-Aldrich, St. Louis, MO, USA), and phosphorylated p38 and total p38 (1:100; Santa Cruz Biotechnology, Dallas, TX, USA). Densitometric analysis of all protein bands was performed using ImageJ software (version 1.50i, National Institutes of Health, Bethesda, MD, USA).

### 2.11. Statistical Analysis

The findings are derived from at least three independent experiments and are presented as the mean ± standard error of the mean (SEM). When statistical analysis was mentioned, group comparisons were performed using ANOVA, followed by Tukey’s HSD post hoc test to determine statistical significance. Differences were considered statistically significant at * *p* < 0.05.

## 3. Results

### 3.1. Spectroscopic Verification of L-PDRN Purity and Electrophoretic Comparison with PDRN

To determine whether PDRN originates from *Lactobacillus*, we conducted a purity test using a spectroscopic method. The final PDRN sample was appropriately diluted for analysis. The absorbance measured at 280 nm was 0.602, and at 260 nm, it was 1.147, resulting in a 260/280 ratio of 1.91. Considering the dilution factor, the final DNA concentration was calculated. The pH of the L-PDRN sample was 6.56, indicating near-neutral conditions suitable for subsequent biological or biochemical applications.

**Figure 1:**
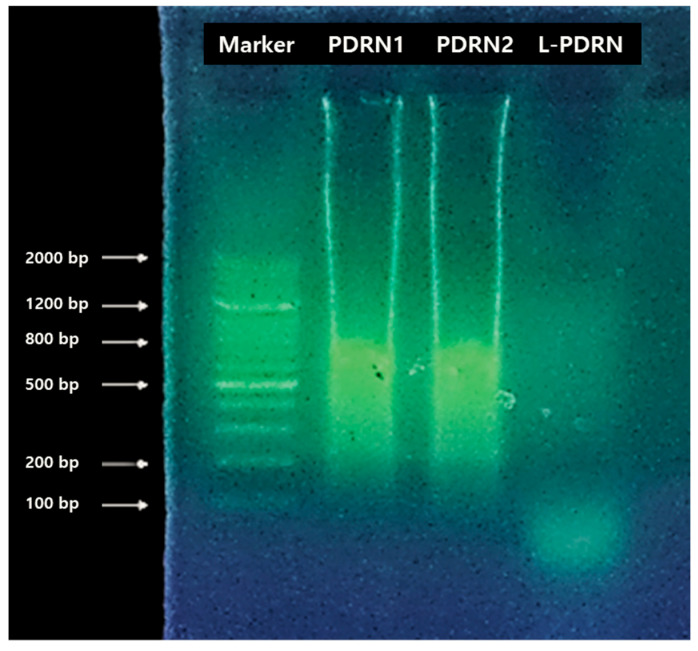
Electrophoretic analysis reveals that L-PDRN comprises smaller DNA fragments (under 100 bp) compared to salmon-derived PDRN (200–800 bp). Using a 100 bp DNA marker, the size difference is clearly observed, with lanes representing salmon PDRN samples (PDRN1 = Sample 1; PDRN2 = Sample 2) and L-PDRN. These findings highlight the unique structural characteristics of L-PDRN, which are further investigated in subsequent bioactivity assays.

Electrophoretic analysis showed that salmon-derived nucleotides primarily contain DNA fragments ranging from 200 to 800 bp, whereas probiotic nucleotides predominantly measure under 100 bp (Figure 1). Because probiotic nucleotides have molecular weights two to eight times lower than those of salmon nucleotides, they are likely to display higher skin absorption rates and superior functional properties compared with salmon-derived nucleotides. However, in the current study, it was only confirmed that the PDRN size is below 100 bp, and the results were limited to a specific strain of *Lactobacillus*. Therefore, differences in PDRN size across various strains remain to be explored in future studies.

### 3.2. Comparison of SOD-like Activity Between PDRN and L-PDRN

**Figure 2:**
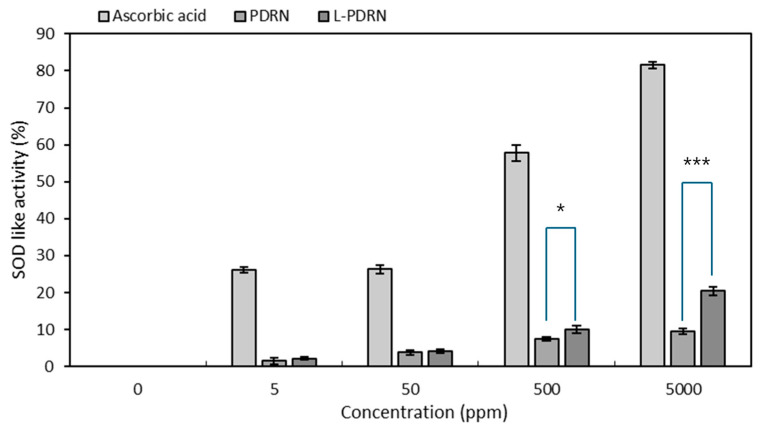
Comparison of SOD-like activity between PDRN and L-PDRN at different concentrations (5, 50, 500, and 5000 ppm). At 500 ppm, L-PDRN exhibited significantly higher SOD-like activity compared to PDRN (* *p* < 0.05). At 5000 ppm, the SOD-like activity of L-PDRN was more than double that of PDRN (*** *p* < 0.001).

Experiments were conducted to evaluate the antioxidant activities of PDRN and L-PDRN by comparing their superoxide dismutase (SOD)-like activities at different concentrations, demonstrating that L-PDRN exhibited significantly higher activity at both concentrations. At 500 ppm, L-PDRN showed a SOD-like activity of 10.02% (±0.96), compared to PDRN’s 7.56% (±0.51), with a statistically significant difference (*p* < 0.05) (Figure 2). At 5000 ppm, L-PDRN’s activity was 20.41% (±1.07), more than double that of PDRN’s 9.42% (±0.80), with a highly significant difference (*p* < 0.001). These results suggest that L-PDRN has enhanced antioxidant potential, especially at higher concentrations. The increased antioxidant activity of L-PDRN may be due to structural or functional modifications that enhance its radical scavenging abilities. This superior performance indicates that L-PDRN could be more effective in applications requiring strong antioxidant effects, such as in high-stress oxidative environments or formulations targeting skin aging or regeneration.

### 3.3. DPPH Radical Scavenging Activity of PDRN and L-PDRN

**Figure 3:**
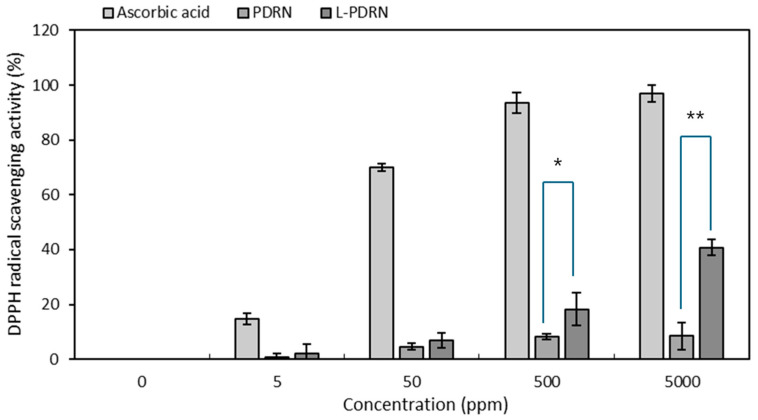
DPPH radical scavenging activity of PDRN and L-PDRN at various concentrations. L-PDRN demonstrated significantly higher DPPH radical scavenging activity compared to PDRN at both 500 ppm (* *p* < 0.05) and 5000 ppm (** *p* < 0.01).

At 500 ppm, L-PDRN exhibited significantly higher DPPH radical scavenging activity (18.28% ± 5.95) compared to PDRN (8.32% ± 0.96), with a notable difference (*p* < 0.05) (Figure 3). At 5000 ppm, L-PDRN’s activity (40.78% ± 2.84) was nearly fivefold greater than PDRN’s (8.45% ± 5.02), demonstrating a highly significant improvement (*p* < 0.01) and reduced variability at higher concentrations. These results indicate that L-PDRN provides substantially enhanced antioxidant capacity over PDRN across both moderate and high concentrations. A study published in *Marine Drugs* investigated the antioxidant activity of PDRN extracted from *Apostichopus japonicus* (AJS-PDRN). The research demonstrated that AJS-PDRN exhibited significant scavenging effects on DPPH radicals, indicating its potent antioxidant properties [16]. This is in line with these results.

Furthermore, referencing established criteria for PDRN substances [45], L-PDRN can be considered a potent PDRN variant suitable for applications requiring strong antioxidant activity compared to PDRN.

### 3.4. Protective Effects of PDRN and L-PDRN on HaCaT Cell Viability Under Oxidative Stress

**Figure 4:**
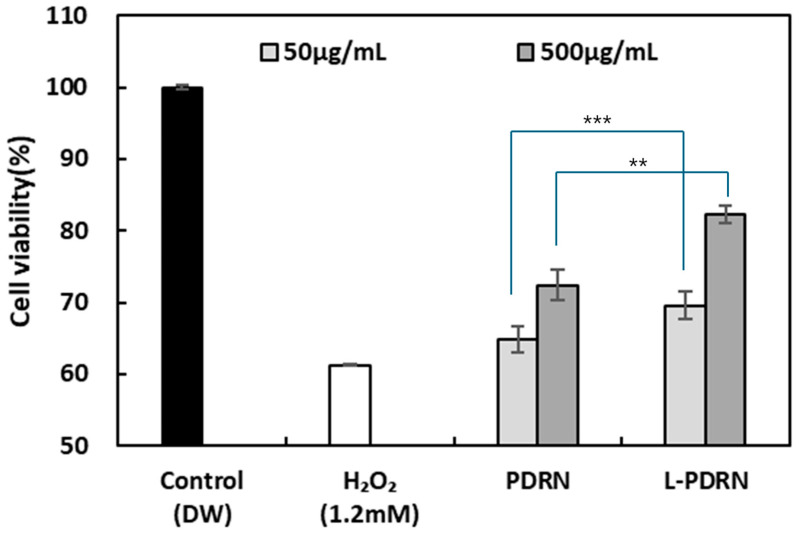
Protective effects of PDRN and L-PDRN on HaCaT cell viability under oxidative stress. HaCaT cell viability was assessed after exposure to 1.2 mM H₂O₂. L-PDRN demonstrated significantly higher viability than PDRN at both 50 μg/mL (*** *p* < 0.001) and 500 μg/mL (** *p* < 0.01). These results highlight the protective effect of L-PDRN against oxidative stress.

To evaluate the protective effects of PDRN and L-PDRN against oxidative stress induced by H_2_O_2_, HaCaT cell viability was assessed. The control group treated with distilled water (DW) showed 100% viability, while exposure to 1.2 mM H_2_O_2_ significantly reduced viability to 61.24% ± 2.06 (Figure 4). In the absence of 1.2 mM H_2_O_2_ treatment, no toxicity was observed for L-PDRN and PDRN at any experimental concentration.

Treatment with PDRN and L-PDRN demonstrated concentration-dependent protective effects. At 50 μg/mL, PDRN increased cell viability to 64.81% ± 1.82, while L-PDRN showed improved viability of 69.58% ± 1.91. The comparison between these groups yielded a statistically significant result (*** *p* < 0.001), indicating the superior protective effect of L-PDRN at this concentration.

At 500 μg/mL, both PDRN and L-PDRN further enhanced cell viability, with PDRN achieving 72.43% ± 2.13 and L-PDRN reaching 82.31% ± 1.24. The comparison between these groups also revealed statistical significance (** *p* < 0.01), confirming the enhanced efficacy of L-PDRN at higher concentrations.

These findings suggest that L-PDRN exhibits a greater ability to mitigate oxidative stress-induced cytotoxicity compared to PDRN. The superior performance of L-PDRN could be attributed to its structural or functional properties, which may enhance its bioavailability or cellular uptake, thus leading to more effective protection of HaCaT cells.

### 3.5. Wound-Healing Effects of PDRN and L-PDRN in HaCaT Cells

TNF-α is a pro-inflammatory cytokine known to disrupt normal cellular functions, including migration and wound healing. For instance, a study investigating the effects of gallic acid on TNF-α-induced MMP-1 expression in HaCaT cells treated the cells with 10 ng/mL TNF-α to induce an inflammatory response. The study found that TNF-α treatment increased MMP-1 expression, which is associated with extracellular matrix degradation and impaired wound healing [46]. Another study examined the inhibitory effects of *Terminalia chebula* on chemokine production in HaCaT cells stimulated with TNF-α and IFN-γ. The researchers used 10 ng/mL concentrations of these cytokines to induce inflammation and observed that this treatment elevated levels of pro-inflammatory chemokines, which can hinder cell migration and wound repair [47].

**Figure 5:**
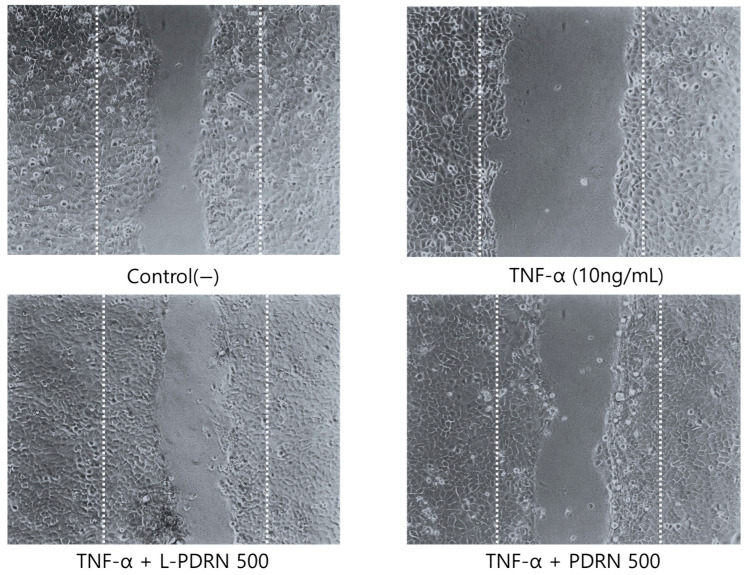
Representative images of wound-healing assay in HaCaT cells at 18 h under different treatments. This figure showcases the wound healing progression in HaCaT cells under various conditions, including control, TNF-α (10 ng/mL), TNF-α + PDRN, and TNF-α + L-PDRN at 500 μg/mL, at 18 h (post-healing). The images highlight the effects of each treatment on the closure of the scratched area, with L-PDRN showing enhanced wound closure compared to PDRN and TNF-α treated group. The area between the two white dotted lines in each image represents the region where cells were initially removed by a scratch.

To compare the wound-healing effects of PDRN and L-PDRN, a wound-healing assay was performed on HaCaT cells to evaluate their impact on cell migration and wound closure over an 18 h period. Initially, the control group exhibited an average wound area of 1,109,534 ± 1542 μm^2^, which naturally decreased to 502,546 ± 3550 μm^2^ after 18 h, reflecting effective inherent wound healing. In contrast, treatment with TNF-α (10 ng/mL) impeded this process, leaving a larger wound area of 1,029,807 ± 13,619 μm^2^, highlighting its inhibitory effect on cell migration and repair (Figure 5).

Both PDRN and L-PDRN treatments significantly enhanced wound healing under inflammatory conditions induced by TNF-α, with L-PDRN demonstrating superior efficacy at both tested concentrations. At a concentration of 500 μg/mL, L-PDRN reduced the wound area to 670,809 ± 1246 μm^2^, outperforming PDRN, which reduced the area to 863,069 ± 6649 μm^2^. These findings highlight the potential of L-PDRN to effectively promote wound healing compared to PDRN in inflammatory environments. While this study focused on conditions with TNF-α to model inflammation, further research is needed to explore the effects of L-PDRN under non-inflammatory conditions, which may provide additional insights into their broader therapeutic potential. However, PDRN seems to be particularly suited for addressing impaired wound healing caused by inflammation rather than general wound healing [48]. We believe this aligns with the underlying mechanism of PDRN.

### 3.6. Effects of PDRN and L-PDRN on NO Production Under Non-Inflammatory and Inflammatory Conditions

Without lipopolysaccharide (LPS) stimulation: In the absence of LPS stimulation, the negative control group (Control−) exhibited minimal NO production at 9.01 ± 0.10 μM, reflecting baseline immune activity. The positive control group (Control+ with LPS) displayed a significant increase in NO levels, reaching 35.34 ± 0.79 μM, confirming its role in immune activation.

**Figure 6:**
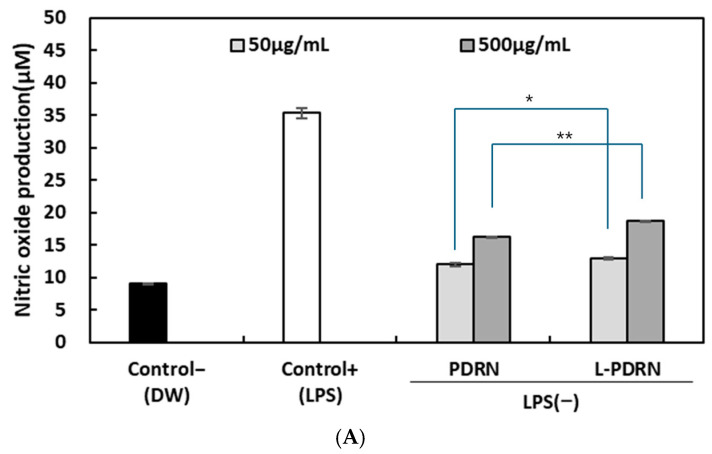

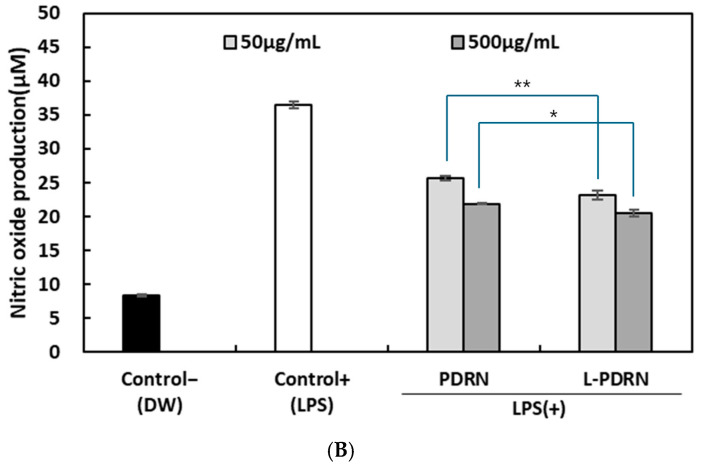
NO production analysis without LPS stimulation (**A**): Comparison of NO production in Raw264.7 cells treated with PDRN and L-PDRN in the absence of LPS. L-PDRN exhibits higher NO levels compared to PDRN, indicating enhanced immunostimulatory effects (* *p* < 0.05, ** *p* < 0.01). NO production analysis with LPS stimulation (**B**)**:** NO production in Raw264.7 cells treated with PDRN and L-PDRN in the presence of LPS. Both compounds reduce NO levels, demonstrating anti-inflammatory properties. Significant differences are noted at 50 μg/mL (** *p* < 0.01) and 500 μg/mL (* *p* < 0.05).

Treatment with PDRN and L-PDRN showed increases in NO production. At 50 μg/mL, PDRN resulted in 11.95 ± 0.21 μM, while L-PDRN yielded 12.88 ± 0.24 μM, showing a statistically significant increase (*p* < 0.05). At 500 μg/mL, PDRN increased NO levels to 16.16 ± 0.13 μM and L-PDRN to 18.67 ± 0.06 μM, with a highly significant difference (*p* < 0.01). These results suggest that L-PDRN may have greater potential to enhance NO production under non-stimulated conditions, warranting further investigation to evaluate its immunomodulatory potential (Figure 6A).

With LPS stimulation: Under LPS stimulation, the negative control group (Control−) produced minimal NO levels at 8.34 ± 0.10 μM, while the positive control group (Control+ with LPS) achieved robust NO production at 36.51 ± 0.54 μM, demonstrating effective immune activation.

Both PDRN and L-PDRN reduced NO production compared to the LPS-only control group, suggesting potential anti-inflammatory effects. At 50 μg/mL, PDRN yielded 25.68 ± 0.35 μM, slightly higher than L-PDRN (23.18 ± 0.67 μM), with statistical significance (*p* < 0.01). At 500 μg/mL, PDRN maintained higher NO levels (21.84 ± 0.10 μM) compared to L-PDRN (20.57 ± 0.50 μM), also showing statistical significance (*p* < 0.05) (Figure 6B).

These findings suggest that PDRN and L-PDRN may have differing roles in modulating NO production. L-PDRN showed increased NO production under non-stimulated conditions, which could indicate a potential to enhance immune activity in specific settings. Moreover, L-PDRN exhibited greater efficacy in reducing NO production under LPS stimulation, suggesting its potential utility in managing inflammatory conditions. The observed differences in bioactivity between PDRN and L-PDRN could be related to structural or functional variations, highlighting the need for further research to clarify their mechanisms of action.

### 3.7. Effects of L-PDRN on FAK-AKT Signaling in HaCaT Cells

To confirm the function of L-PDRN as an enhanced type of PDRN, Western blot experiments were performed. Previous studies have shown that PDRN promotes the phosphorylation of FAK through the A2A receptor signaling pathway, which is critical for cell proliferation and barrier improvement [14]. The researchers found that Panax PDRN acts as an agonist of the A2A receptor, leading to increased phosphorylation of focal adhesion kinase (FAK) and protein kinase B (AKT). This activation promotes cell proliferation and migration, contributing to wound healing and improved skin barrier function.

**Figure 7:**
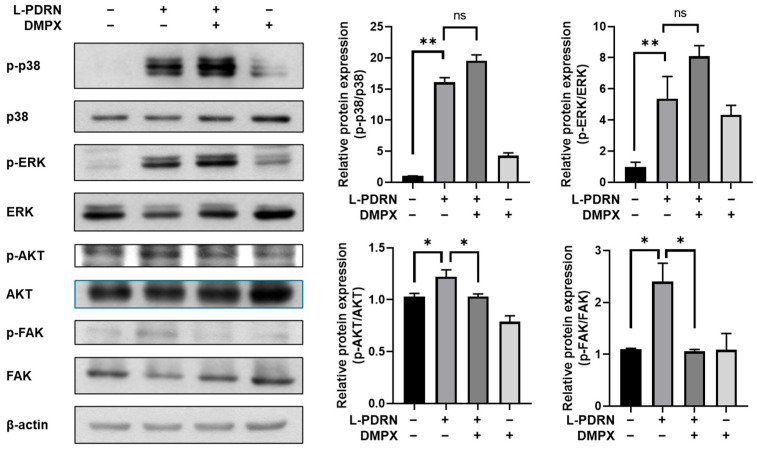
Representative Western blot images and corresponding densitometric analysis in HaCaT cells. Cells were treated with 10 nM DMPX and 500 μg/mL L-PDRN for 60 min. Phosphorylation levels of FAK, AKT, ERK, and p38 were assessed via Western blotting. The bar graph on the right illustrates the densitometric analysis of the Western blot bands. Statistical significance between groups is indicated as follows: ** *p* < 0.01, * *p* < 0.05, and ns (not significant).

In this study, we investigated the effect of L-PDRN by examining the phosphorylation levels of FAK and AKT. The results demonstrated that L-PDRN significantly increased the phosphorylation levels of FAK and AKT (Figure 7). Moreover, treatment with DMPX, a specific inhibitor of FAK phosphorylation, effectively reduced L-PDRN-induced phosphorylation levels of FAK and AKT, confirming that L-PDRN activates the FAK-AKT signaling pathway, similar to PDRN. PDRN promotes cell proliferation through the activation of the A2A receptor, leading to increased phosphorylation of FAK and AKT. This effect was inhibited by DMPX, an A2A receptor antagonist, indicating that PDRN’s action is mediated via the A2A receptor [7]. Similarly, PDRN’s therapeutic effects were attenuated by DMPX, further confirming the involvement of the A2A receptor in mediating PDRN’s actions [2].

Interestingly, L-PDRN also increased the phosphorylation levels of p38 and ERK, downstream molecules associated with the FAK-AKT pathway. However, these effects were not attenuated by DMPX, indicating that L-PDRN-mediated activation of p38 and ERK is independent of FAK. Some studies reported that PDRN can either activate or inhibit the ERK pathway in human skin cells. These effects vary depending on the cell type, suggesting that PDRN may act through mechanisms independent of the A2A receptor [49]. The other study demonstrated that PDRN helps alleviate voiding dysfunction caused by interstitial cystitis by suppressing inflammation and apoptosis. While this effect is mainly attributed to A2A receptor activity, the findings also imply that PDRN may act through other pathways [2].

These findings collectively confirm that L-PDRN possesses the fundamental functionalities of PDRN, including activation of the FAK-AKT signaling pathway through the A2A receptor. Furthermore, L-PDRN demonstrates enhanced signaling versatility by engaging alternative pathways for the activation of p38 and ERK, which are not mediated by FAK. This enhanced capability positions L-PDRN as a more powerful and multifaceted type of PDRN, expanding its potential applications in cell proliferation, barrier improvement, and skin regeneration compared to PDRN.

## 4. Discussion

This study marks a significant milestone as the first to explore microbial, *Lactobacillus*-derived, or biome-sourced PDRN—specifically L-PDRN—and its potential applications. Traditionally, PDRN has been extracted from salmonid gonads, which are constrained by seasonal availability, high production costs, and environmental sustainability issues. In response to these challenges, alternative sources such as plants and marine organisms have been investigated. Beneficial microbial sources, or probiotics, are microorganisms that promote health in the human body, with many originating from the *Lactobacillus* genus. They are well-known for their health-enhancing effects achieved through various mechanisms [50]. However, microbial sources, particularly probiotics like *Lactobacillus rhamnosus*, present unique advantages, including year-round availability, rapid growth, and a reduced environmental footprint. Notably, *Lactobacillus rhamnosus* can be produced at high concentrations of 10^10^–10^11^ CFU/g, demonstrating its potential for mass production [51]. Moreover, *Lactobacillus* strains are generally recognized as safe (GRAS), have well-established culture methods [52], and are easy to acquire in large quantities, making them a sustainable and innovative solution for PDRN production. *Lactobacillus* strains are widely used in the manufacturing of fermented foods, including dairy, meat, vegetables, and sourdough breads [53]. This widespread use in food production indicates their availability in large quantities. The key findings and significance will be detailed in the next section.

Structural superiority of L-PDRN: The electrophoretic analysis highlighted that L-PDRN consists of smaller DNA fragments (under 100 bp) compared to traditional salmon-derived PDRN (200–800 bp). This smaller molecular size has the potential to improve skin absorption rates, thereby enhancing its bioavailability and functional properties. Studies have shown that smaller DNA molecules are more effective at penetrating the skin [54]. Smaller oligonucleotides are notably more effective at enhancing skin absorption, as molecular size plays a critical role in skin penetration. Generally, smaller molecules have a distinct advantage in permeating through the skin layers [55]. This structural advantage positions L-PDRN as a superior alternative, particularly for applications requiring high bioactivity, such as skin regeneration and wound healing, especially in topical formulations like cosmetics or ointments.

Enhanced antioxidant activity: This study evaluated the biological effects of PDRN on various cellular activities [56]. PDRN exhibits dose-dependent antioxidant activity, effectively reducing oxidative stress in skin cells and making it beneficial for combating cellular damage caused by free radicals [56]. The findings revealed that PDRN displayed dose-dependent antioxidant activities in a radical scavenging assay, effectively suppressing oxidative stress in skin cells. L-PDRN demonstrated significantly higher antioxidant activity in both SOD-like and DPPH assays compared to PDRN, particularly at higher concentrations. This suggests that L-PDRN is better equipped to combat oxidative stress, which is critical for applications like skin aging prevention and tissue repair. The structural modifications in L-PDRN may contribute to its enhanced radical scavenging efficiency, making it a strong candidate for oxidative stress-related therapies.

Regenerative and wound-healing potential in inflammatory conditions: The wound-healing assay demonstrated that L-PDRN was more effective than PDRN in promoting cell migration and wound closure in HaCaT cells under pro-inflammatory conditions induced by TNF-α. At a concentration of 500 μg/mL, L-PDRN resulted in a significantly smaller wound area compared to PDRN, highlighting its superior efficacy. These results suggest that L-PDRN holds significant potential for tissue regeneration and wound healing, likely due to its enhanced interaction with cellular repair pathways under inflammatory conditions like those targeted by PDRN.

Potential for immunomodulatory effects: L-PDRN exhibited a potential for immunomodulatory properties by modestly enhancing NO production in Raw264.7 cells under non-inflammatory conditions, while PDRN demonstrated slightly greater effectiveness in reducing inflammation under LPS-induced conditions. These findings suggest complementary roles, with L-PDRN possibly supporting immune activity. This indicates that L-PDRN may possess immunomodulatory capabilities.

Unique mechanisms of action: Western blot analysis revealed that L-PDRN activates the FAK-AKT signaling pathway via the A2A receptor, like PDRN. However, L-PDRN uniquely phosphorylates p38 and ERK independently of FAK, indicating additional pathways for cellular modulation. This signaling versatility further distinguishes L-PDRN, expanding its potential applications in skin barrier improvement and cell proliferation.

Implications of the first report: This study not only introduces a novel and sustainable source of PDRN but also underscores the broader implications of microbial-derived PDRN—specifically L-PDRN—in advancing biomedical applications. By leveraging probiotics such as *Lactobacillus rhamnosus*, this research highlights the feasibility of producing PDRN through environmentally friendly and scalable methods. The GRAS status of *Lactobacillus* strains, coupled with well-characterized cultivation techniques and straightforward biomass acquisition, further strengthens the case for microbial-derived PDRN. The findings pave the way for further exploration of microbial sources for PDRN production, positioning L-PDRN as a superior and versatile biomaterial for diverse therapeutic applications, including oxidative stress management, wound healing, and immune modulation.

## 5. Conclusions

This study introduces *Lactobacillus*-derived PDRN (L-PDRN) as an alternative to traditional salmon-derived PDRN. The key findings emphasize the structural and functional superiority of L-PDRN, highlighting its smaller DNA fragment size (under 100 bp), which enhances skin absorption and bioavailability, making it particularly effective for skin regeneration and wound healing. This study demonstrates that L-PDRN has enhanced antioxidant activity, surpassing traditional PDRN in reducing oxidative stress, a critical factor in combating skin aging and cellular damage. Additionally, the regenerative potential of L-PDRN is evident in its superior ability to promote cell migration and wound closure under inflammatory conditions, showcasing its efficacy in tissue repair. The unique signaling mechanisms of L-PDRN, including activation of the FAK-AKT pathway and independent phosphorylation of p38 and ERK, expand its therapeutic potential, especially in immune modulation and cellular repair. Importantly, the use of *Lactobacillus rhamnosus* as a microbial source offers significant advantages over traditional methods, including year-round availability, sustainability, and reduced environmental impact. The GRAS status and ease of cultivation further solidify its practicality for large-scale production. In summary, L-PDRN represents a sustainable, versatile, and highly effective biomaterial with applications in skin care, wound healing, oxidative stress management, and immune modulation. This research lays the foundation for further exploration of microbial sources for PDRN production and broadens its potential in biomedical applications. Future studies are needed to investigate wound healing under non-inflammatory conditions, validate the precise immunomodulatory capabilities, and compare the PDRN sizes across different microbial strains. Nevertheless, this study is significant as the first to propose the potential of microbial-derived PDRN, and the findings pave the way for further exciting research in this field.

## Data Availability

The data that support the findings of this study are available from the corresponding author upon reasonable request.
